# A case report: identification of a novel exon 1 deletion mutation in the *GNE* gene in a Chinese patient with GNE myopathy

**DOI:** 10.1097/MD.0000000000022663

**Published:** 2020-10-09

**Authors:** Jing Miao, Xiao-jing Wei, Xu Wang, Xiang Yin, Xue-fan Yu

**Affiliations:** Department of Neurology and Neuroscience Center, the First Affiliated Hospital of Jilin University, Changchun, Jilin, People's Republic of China.

**Keywords:** case report, distal myopathy with rimmed vacuoles, *GNE* gene, myopathy

## Abstract

**Rationale:**

GNE myopathy is caused by mutations in the UDP-*N*-acetylglucosamine-2-epimerase/*N*-acetylmannosamine kinase(*GNE*) gene and is clinically characterized by progressive weakness and atrophy of the lower-limb muscles with quadriceps sparing. Nearly all *GNE* mutations that have been reported thus far in various ethnic populations around the world have been missense or nonsense mutations.

**Patient concerns:**

We describe the case of a 32-year-old woman with GNE myopathy. The patient presented with progressive weakness of the lower-limb muscles that had spread to her legs. Her serum creatine kinase level was higher than the normal range. Mild myogenic changes were detected in the tibialis anterior muscles on electromyography, and moderate fatty infiltration was observed in various lower-limb muscles on magnetic resonance imaging. Histopathological examination of a skeletal muscle biopsy specimen revealed variation in muscle fiber size, rimmed vacuoles, and disorganized intermyofibrillar networks. DNA sequencing testing revealed a compound heterozygous mutation consisting of a known mutation (c.620A > T in exon 3) and a novel (exon 1 deletion) mutation.

**Diagnoses:**

Taken together, the clinical features, laboratory testing and DNA findings eventually made the diagnosis of GNE myopathy.

**Interventions and outcomes:**

Based on the diagnosis of the GNE myopathy, the patient was administered sialic acid 6 g a day for 1 year, and up to now, her symptoms did not progress further.

**Lessons:**

We have reported the case of a GNE myopathy patient with compound heterozygous *GNE* gene mutations. This case expands the genotypic spectrum of GNE myopathy.

## 1 Introduction

GNE myopathy (also known as distal myopathy with rimmed vacuoles or hereditary inclusion body myopathy) is caused by mutations in the *GNE* gene. This condition has an autosomal recessive inheritance and is clinically characterized by progressive weakness and atrophy of the lower-limb muscles, with quadriceps sparing, that eventually results in wheelchair dependence within 10 to 20 years.^[1]^ The prevalence of GNE myopathy is estimated to be 4–21/1,000,000.^[1]^ Generally, histopathological examination reveals muscle atrophy associated with rimmed vacuoles and varying muscle fiber sizes. The *GNE* gene is located in the 9p13.3 chromosome band and includes 13 coding exons. Eight different GNE mRNAs encode 8 protein isoforms.^[2]^ The hGNE2 isoform spans 753 amino acids and is encoded by the longest GNE mRNA transcript. To the best of our knowledge, nearly all *GNE* mutations that have been reported thus far in various ethnic populations around the world have been missense or nonsense mutations. The p.V572L and p.D176 V mutations are common in Japanese patients,^[3]^ while the p.L508S, p.D176 V, and p.A631 V mutations are common in Chinese patients.^[4]^ Here, we report the case of a Chinese patient with GNE myopathy caused by a rare compound heterozygous mutation in the *GNE* gene.

## 2 Case presentation

Our patient was a 32-year-old woman with progressively worsening lower-limb muscle weakness over a 3-year period that resulted in an impaired ability to walk on her heels. At the time of presentation to our hospital, the muscle weakness had spread to her legs, causing difficulty in rapid walking and climbing stairs. She also reported experiencing lumbar back pain. She had been born at full term to healthy non-consanguineous parents. Muscle weakness was symmetrically detected on ankle dorsiflexion (MRC 2/5), hip flexion (MRC 4/5), knee extension (MRC 4/5), and knee flexion (MRC 3/5). The deep tendon reflexes were absent. There was no cranial nerve dysfunction or sensory disturbance. The serum creatine kinase level was double the upper limit of the normal range. Needle electromyography revealed mild myogenic changes in the tibialis muscles. magnetic resonance imaging (MRI) examinations were performed on a 3.0T (Magnetom Expert, Siemens, Erlangen, Germany) scanner at the level of the thigh and lower leg muscles. The following sequences were used:

(1) axial T1-weighted fast spin-echo series for fatty infiltration and
(2) axial T2-weighted short-time inversion recovery series with fat suppression series for edema.

**Figure 1:**
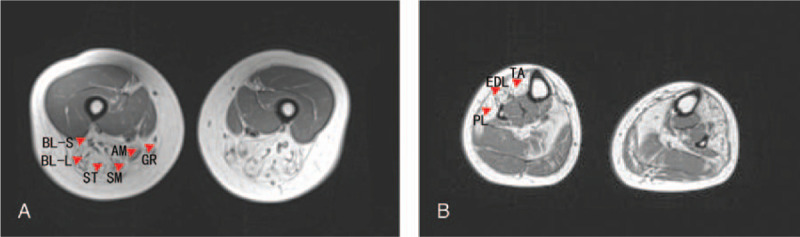
Axial-T1-weighted magnetic resonance images of the muscles showing a typical pattern of muscle involvement. The hyperintense areas reflect fatty infiltration. (A) In the thigh, the long and short head of the biceps femoris (BL-L, BL-S), semimembranosus (SM), semitendinosus (ST), adductor magnus (AM), and gracilis (GR) show moderate fatty infiltration. (B) In the lower leg, the tibialis anterior (TA), extensor digitorum longus (EDL), peroneus longus (PL) show conspicuous fatty changes (red arrows).

**Figure 2:**
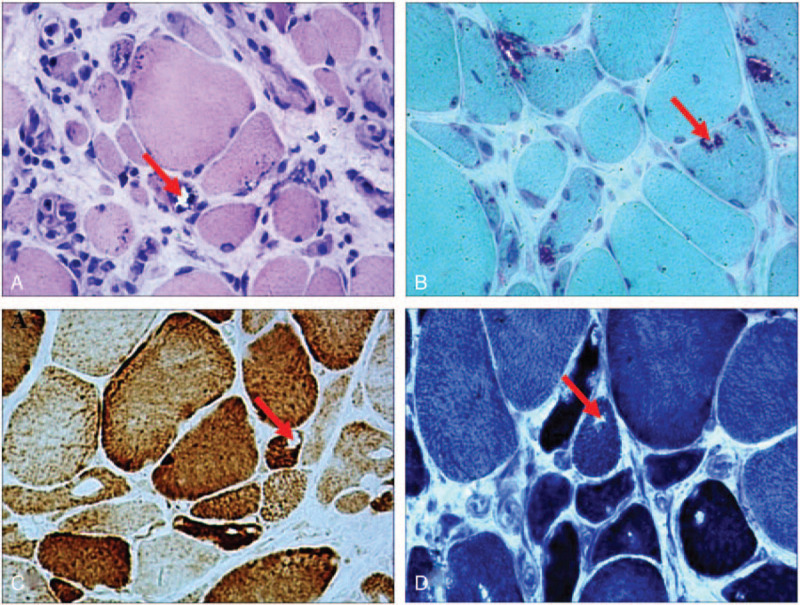
Histopathological examination of the skeletal muscles. (A) Hematoxylin and eosin staining shows muscle fibers of variable sizes and rimmed vacuoles (red arrow). (B) Modified Gomori trichrome staining shows rimmed vacuoles in the muscle fibers (red arrow). Staining for (C) cytochrome oxidase and (D) NADH-tetrazolium reductase shows some fibers with areas lacking enzyme reactivity. (red arrow). (magnification, × 200).

**Figure 3:**
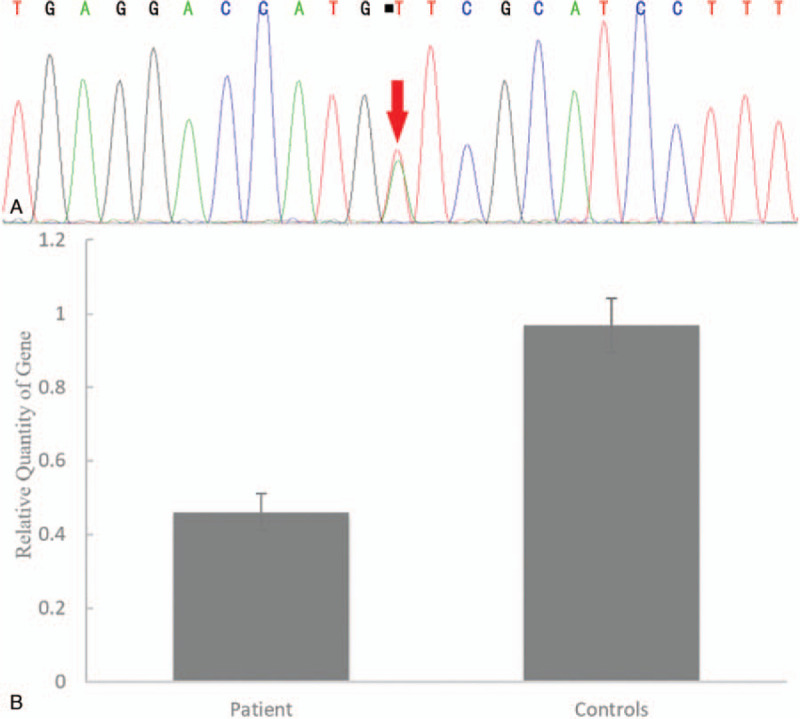
DNA sequencing analysis. (A) Sanger sequence analysis shows a known mutation c.620A > T in exon 3 (red arrow), resulting in the substitution of aspartic acid with valine. (B) Real-time quantitative PCR sequence analysis shows exon 1 content in the patient was reduced to 48% compared with the controls. (mean calculation ratio: 0.46 ± 0.05; control results: 0.96 ± 0.07). PCR = polymerase chain reaction.

Fatty infiltration was detected in the following lower-limb muscles on MRI: the long and short head of the biceps femoris, semimembranosus, semitendinosus, adductor magnus and gracilis muscles in the thigh, and the tibialis anterior, extensor digitorum longus, peroneus longus muscles in the distal leg (Fig. 1A-B). Written informed consent was obtained from the patient, and a skeletal muscle biopsy was taken from the left tibialis anterior muscle. Hematoxylin-eosin staining of the biopsy specimen showed wide variations in fiber size, fiber splitting, and increased interstitial fibrosis. Muscle fiber necrosis and phagocytosis were also observed. Modified Gomori trichrome staining revealed rimmed vacuoles without any ragged red fibers or nemaline rods. Staining for NADH-tetrazolium reductase revealed disorganized intermyofibrillar networks. Cytochrome c oxidase staining showed some fibers with areas lacking enzyme reactivity (Fig. 2A-D). Mutations in the *GNE* gene were analyzed using targeted next-generation sequencing. NM_001128227 was used as a transcript reference sequence. Nucleotide alternations of genomic sequence were confirmed by Sanger sequencing. The copy number variants were analyzed with XHMM(v1.0) and CODEX tools. Copy number variants were detected using a real-time polymerase chain reaction detection system v2.4. The *GNE* gene and the *GAPDH* gene were used as the amplification targets for the quantification of the genome DNA. For the *GNE* gene, the following primers were used: forward, 5′-TCCTCTTTGTAATTTTCCTTTTTCC-3′ and reverse, 5′-GAAAACAGCTGCTCTgCTCATTA-3′. For the *GAPDH* gene, the following primers were used: forward, 5′-CAGGTGGAGCGAGGCTAG-3′ and reverse, 3′-CTACCCATGACTCAGCTTCTCC-5′. The segregation analyses of the mutations were confirmed in the healthy parents. Additionally, we searched the gene bank (HGMD Pro, Pubmed, 1000Genomes, dbSNP and clinvar databases). Finally, we confirmed a known mutation c.620A > T (p.Asp207Val) in exon 3 by Sanger sequencing, which was inherited from the father (Fig. 3A). Real-time quantitative polymerase chain reaction sequence analysis showed exon 1 content in the patient was reduced to 48% compared with the controls (patient: controls = 0.46 ± 0.05: 0.96 ± 0.07), which was novel and inherited from the mother (Fig. 3B). The patient was administered sialic acid 6 g a day for 1 year, and up to now, her symptoms did not progress further.

## 3 Discussions

GNE myopathy is diagnosed on the basis of clinical findings, such as the results of muscle imaging studies and histopathological examination, and is confirmed by genetic studies. The clinical presentation of the patient in this study, including the adult onset of the disease, weakness and atrophy of the lower limbs with quadriceps sparing, and the characteristic muscle histological findings together with the *GNE* gene mutations, was consistent with a diagnosis of GNE myopathy. The *GNE* gene encodes a bifunctional enzyme (UDP-*N*-acetylglucosamine 2-epimerase/*N*-acetylmannosamine kinase) that initiates and regulates the biosynthesis of *N*-acetylneuraminic acid. This compound is the precursor of sialic acids, which are present as sugars in glycoproteins and glycolipids.^[1]^ Thus, sialic acid replacement can be useful in the treatment of patients with GNE myopathy.

MRI pattern is a useful tool in diagnosing muscle disorders. Bugiardini E et al reported that a prominent involvement of the short head of the biceps femoris muscle was a key feature in GNE myopathy.^[5]^ The involvement of the special muscle of our patient is consistent with the observations in other patients with GNE myopathy. However, in addition to the short head of the biceps femoris, the other posterior thigh muscles of the patient were also involved due to the advanced stage of the disease.

Our patient harbors compound heterozygous *GNE* gene mutations in the epimerase domains: c.620A > T (p.Asp207Val) on exon 3 and deletion of exon 1. Interestingly, although c.620A > T is a common mutation in Japanese patients, it is also present in Korean and Chinese patients.^[3,6,7]^ This indicates that this mutation may be an ethnic GNE founder mutation.^[8]^ To the best of our knowledge, exon 1 deletion has not been reported elsewhere. Three larger indels have been reported previously: a 10-bp insertion in exon 3, exon 3 deletion, and a deletion spanning exons 1–9.^[3,9,10]^ All of these variants may result in a termination codon and/or nonsense-mediated decay. The novel exon 1 deletion that was identified in the *GNE* gene in our patient is probably pathogenic for the following reasons:

(1) the mutation was not detected in 200 ethnically matched control chromosomes;
(2) the mutation is crucial for encoding the additional *N*-terminal sequence of hGNE2;^[11]^
(3) exon 1 contains the start codon; therefore, the deletion of exon 1 may affect the amino acid sequence.

The variation in fiber size and the presence of disorganized intermyofibrillar networks in the abnormal fiber regions in our patient, who also harbored a different mutation in the epimerase domain, indicate that these mutations may cause structural and functional changes in the muscle fibers.^[12]^ The presence of rimmed vacuoles and dystrophic fibers in our patient is consistent with the observations in other patients with GNE myopathy and in Gne(-/-)hGNED176VTg mice, indicating that these mutations lead to a decrease in sialic acid production and protein misfolding, which leads to a reduction in the efficiency of both the ubiquitin-proteasome system and autophagy system to degrade protein aggregates.^[13–16]^ All of these events ultimately cause muscle weakness.

The c.620A > T mutation inherited from the father was reported in Japanese, Korean and Chinese patients. Compound with the novel maternal deletion mutation, the paternal mutation resulted in severe phenotypes in our patient. To the best of our knowledge, our patient is the first case reported to feature exon 1 deletion mutation in the epimerase domain. Therefore, this case report not only expands the *GNE* gene mutation spectrum, but also helps to explore the pathogenesis of mutations in epimerase domain. In addition, it is very important to consider GNE myopathy as a differential diagnosis for patients with slowly progressive distal myopathies.

## Acknowledgment

The authors thank to Medjaden Bioscience Limited in proofreading the manuscript.

## Author contributions

Jing Miao drafted the manuscript and figures; Xiao-jing Wei analyzed and interpreted histological data; Xu Wang and Xiang Yin designed and analyzed the study; Xue-fan Yu revised the manuscript and gave the final approval of the version to be published. All authors read and approved the contents of the case report. Jing Miao and Xiao-jing Wei contributed equally to the manuscript.

## Abbreviations

Abbreviation: MRI = magnetic resonance imaging.
